# Retrospective cohort analysis of outpatient antibiotic prescribing for community-acquired pneumonia in Canadian older adults

**DOI:** 10.1371/journal.pone.0292899

**Published:** 2023-10-13

**Authors:** Ariana Saatchi, Jennifer N. Reid, Salimah Z. Shariff, Marcus Povitz, Michael Silverman, David M. Patrick, Andrew M. Morris, James McCormack, Manon R. Haverkate, Fawziah Marra

**Affiliations:** 1 Faculty of Pharmaceutical Sciences, University of British Columbia, Vancouver, British Columbia, Canada; 2 ICES Western, Lawson Health Research Institute, London Health Sciences Centre, London, Ontario, Canada; 3 Department of Medicine, University of Calgary, Calgary, Alberta, Canada; 4 Faculty of Medicine, Western University, London, Ontario, Canada; 5 British Columbia Centre for Disease Control, Vancouver, British Columbia, Canada; 6 School of Population and Public Health, University of British Columbia, Vancouver, British Columbia, Canada; 7 Sinai Health System, University Health Network and University of Toronto, Toronto, Ontario, Canada; HJF: Henry M Jackson Foundation for the Advancement of Military Medicine Inc, NIGERIA

## Abstract

**Background:**

This retrospective cohort study is the first in North America to examine population-level appropriate antibiotic use for community-acquired pneumonia (CAP) in older adults, by agent, dose and duration. With the highest rates of CAP reported in the elderly populations, appropriate antibiotic use is essential to improve clinical outcomes. Given the ongoing crisis of antimicrobial resistance, understanding inappropriate antibiotic prescribing is integral to direct community stewardship efforts.

**Methods:**

All outpatient primary care visits for CAP (aged ≥65 years) were identified using physician billing codes between January 1 2014 to December 31 2018 in British Columbia (BC) and Ontario (ON). Categories of prescribing were derived from existing literature, and constructed for clinical relevance using Canadian and international guidelines available during the study period. Categories were mutually exclusive and included: guideline adherent (first-line agent, adherent dose/duration), clinically appropriate (non-first line agent, presence of comorbidities), effective but unnecessary (first-line agent, excess dose/duration), undertreatment (first-line agent, subtherapeutic dose/duration), and not recommended (non-first line agent, absence of comorbidities). Proportions of prescribing were examined by category. Temporal trends in prescribing were examined using Poisson regression.

**Results:**

A total of 436,441 episodes of CAP were identified, with 46% prescribed an antibiotic in BC, and 52% in Ontario. Guideline adherent prescribing was minimal for both provinces (BC: 2%; ON: 1%) however the largest magnitude of increase was reported in this category by the final study year (BC—Rate Ratio [RR]: 3.4, 95% Confidence Interval [CI]: 2.7–4.3; ON—RR: 4.62, 95% CI: 3.4–6.5). Clinically appropriate prescribing accounted for the most antibiotics issued, across all study years (BC: 61%; ON: 74%) (BC—RR: 0.8, 95% CI: 0.8–0.8; ON—RR: 0.9, 95% CI: 0.8–0.9). Excess duration of therapy was the hallmark characteristic for effective but unnecessary prescribing (BC: 92%; ON: 99%). The most common duration prescribed was 7 days, followed by 10. Not recommended prescribing was minimal in both provinces (BC: 4%; ON: 7%) and remained stable by the final study year (BC—RR: 1.1, 95% CI: 0.9–1.2; ON—RR: 0.9, 95% CI: 0.9–1.1).

**Conclusion:**

Three quarters of antibiotic prescribing for CAP was appropriate in Ontario, but only two thirds in BC. Shortening durations—in line with evidence for 3 to 5-day treatment presents a focused target for stewardship efforts.

## Introduction

Community-acquired pneumonia (CAP) is a leading cause of mortality and morbidity for older adults. The most commonly identified pathogens include *Streptococcus pneumoniae*, as well as respiratory viruses [1, 2]. Despite microbiologic testing, no pathogen is detected in up to 62% of inpatient cases [1, 3, 4]. Deteriorations in functional status following illness occurs in 10% of patients post-recovery, jeopardizing quality of life [5]. Moreover, despite the polysaccharide pneumococcal vaccine being available in Canada through publicly-funded immunization programs since the 1990s, annual rates of CAP have continued to rise, with 212 additional incident cases per 100,000 in the province of British Columbia (BC) by 2018 (compared to 2010), and 239 in Ontario (ON) [6]. Highest infection rates continue to be reported in the elderly populations [7]. Adherence to clinical guidelines in antibiotic treatment selection has been associated with improved patient outcomes, and reduced healthcare costs [8–10]. Given the elevated risk of mortality in this population, understanding appropriate antibiotic use is essential to protect this vulnerable population.

The etiology of CAP is not homogenous [6]. However, unlike the case of viral respiratory tract infections, delays in antibiotic treatment for CAP increase the risks for adverse outcomes [9, 11, 12]. As such, the goals of antimicrobial stewardship for CAP include the optimization, rather than reduction, of empiric antibiotic prescribing. The evaluation of antibiotic prescribing is often contingent on patient factors not available within routine, administrative health data, as such, appropriate outpatient use has been examined in limited scope [13]. Efforts to evaluate prescribing quality are further limited by the absence of standardized nomenclature [14]. In 2018, a Canadian antimicrobial stewardship program published categories of antibiotic prescribing aimed to address aforementioned limitations; provide more clinically relevant and granular interpretations of prescribing quality; and offer a nuanced lexicon with which to engage [15].

This retrospective cohort study is the first in North America to examine if antibiotic prescribing was appropriate for CAP in older adults, by agent, dose and duration. The use of clinical practice guidelines to define discrete categories of prescribing quality offer a novel, more objective interpretation of appropriate prescribing, when compared to previous efforts of expert opinion elicitation. Our objectives were to determine the quality of empiric antibiotic prescribing for CAP, across two Canadian provinces, and expand interpretations of appropriate prescribing by agent, dose, and duration.

## Methods and materials

### Data sources

This study was approved by the Behavioural Research Ethics Board of the University of British Columbia (H19-00799). All methods were carried out in accordance with relevant guidelines and regulations. Data was extracted, anonymized, and made available to researchers by Population Data BC. Participant informed consent was waived by IRB approval. Written consent for publication was obtained from the BC Ministry of Health and Population Data BC.

The BC Ministry of Health and ICES (formerly known as the Institute for Clinical Evaluative Sciences) in Ontario house several health care-related databases, which contain comprehensive information on their respective populations. The BC Medical Services Plan (MSP) and Ontario Health Insurance Plan (OHIP) record all claims submitted by physicians, including diagnostic codes [16]. Antibiotic dispensation data were available through BC PharmaNet, and Ontario Drug Benefit (ODB) program systems [17, 18]. Patient demographics, including age and sex, were extracted from a Consolidation file in BC, and the Registered Persons Database (RPDB) in Ontario [19]. Further patient information including comorbidity data, were identified through the Discharge Abstract Database (DAD) for both provinces, with additional ICES-validated subsets utilized for specific conditions [20].

### Study population & case definitions

Our study included all residents of BC and Ontario with a physician record (family and/or general practitioner) for CAP, from January 1, 2014 to December 31, 2018. Patients aged <65 years, and those living in long-term care facilities were excluded. Physician records were identified using relevant ICD-9 (480–481) and OHIP (486; 986) codes for pneumonia. Acute episodes of infection were defined using a 14-day window with all recurrent physician visits within 14 days flagged as a single episode of infection. The index date was identified as the first physician visit within an episode. Chronic CAP episodes which exceeded 30 days in total length were excluded. As hospital dispensation data were not available for this study, any patients admitted to hospital up to 5 days following the end of an episode were excluded, on an assumption that they were treated for CAP as an inpatient. These criteria have previously been used to identify episodes of infection in Canadian outpatient care [21].

A prescription was linked to an episode of CAP using an algorithm that matched the date on which the medication was dispensed to a corresponding physician visit within a permissible period of time. This linkage window ranged from episode index date until the final physician visit within an episode, with an additional 5-day follow-up. If multiple prescriptions were present within a given linkage period, only the first dispensation record was kept to evaluate empiric prescribing. All cells with n < 6 were excluded from analyses to preserve subject anonymity.

### Clinical guidelines

A literature review identified relevant CAP guidelines and prescribing resources for Canadian outpatient care. Clinical practice guidelines and treatment recommendations were pulled from the following resources: American Thoracic Society (ATS), Infectious Disease Society of America (IDSA), National Institute for Health and Care Excellence (NICE), British Thoracic Society (BTS), and Bugs & Drugs: a provincial reference [22–26]. Guidelines were reviewed and first-line agent(s), dosing and duration recommendations were extracted. First-line agents for the treatment of CAP were identified as: oral amoxicillin [1g BID *or* TID x 5 days], amoxicillin clavulanate [875mg BID x 5 days], and doxycycline [200mg day 1; 100mg BID day(s) 2–5]. Given variability in recommended dosing, both lower- and upper-limit bounds were calculated for each agent to identify the permissible range for guideline concordant average daily dose. Across relevant references, recommended durations of treatment for outpatient, non-severe CAP specified 5 days of therapy (strong recommendation; moderate quality of evidence). Preceding, and throughout the study period, shorter-course treatments had been examined for effectiveness, and non-inferiority, across several trials [27–32]. Given the existing body of literature, and the cohesion of guideline recommendations—5 days was selected as the appropriate duration.

### Categories of prescribing quality

Utilizing the criteria and lexicon put forth by Dresser *et al*. (2018), study categories of prescribing quality were mutually exclusive, and included: (1) guideline adherent; (2) clinically appropriate; (3) undertreatment; (4) effective but unnecessary; and (5) not recommended (S1 Table). Guideline adherent: prescription of a first-line agent with guideline concordant dose *and* duration. Clinically appropriate: use of non-first line antibiotic agents, of any dose or duration, in the presence of *at least one* patient clinical justification. Patient justifications include: relevant comorbidities (e.g., COPD, cancer, myocardial infarction, etc.; complete overview of patient factors can be viewed in S2 Table), drug interactions, immunosuppressant medications, and Charlson index (moderate/severe) score. Patient comorbidities were identified using a breadth of outpatient (ICD-9/OHIP), hospital (ICD-10), as well as dispensation records (PharmaNet/ODB) in order to satisfy previously validated ICES case definitions. Under treatment: first-line agent that can be improved in *at least one* of the following categories: dose (i.e. < recommended), and/or duration (i.e. < recommended). Effective but unnecessary: first-line agent that can be improved in *at least one* of the following categories: dose (i.e. > recommended), and/or duration (i.e. > recommended). Not recommended: use of non-first line antibiotic agents, of any dose and duration, in the *absence of all* patient justifications. If dose and duration deviated in opposing directions a prescription was assigned to the category of undertreatment as sub-therapeutic utilizations were hypothesized to be of higher clinical significance. As laboratory data were not available to verify patient creatine clearance, undertreatment prescriptions in the presence of patient renal dysfunction (e.g., chronic kidney disease) were re-classified as clinically appropriate, in line with renal dosing adjustments.

### Outcomes & statistical analyses

Baseline cohort characteristics were examined by age, sex, income quintile and rurality, with the first CAP record utilized as index per patient. Patient comorbidities, presence of immunosuppressant medications and/or drug interactions, as well as Charlson score were also examined, by episode of CAP. Primary outcomes included: the proportion of total CAP episodes prescribed as well as the proportion of antibiotic prescribing by category of quality; results were then stratified by age and sex. Rates of prescribing were calculated as the number of prescriptions per 1000 population, using cohort-specific denominators per province. Temporal trends in prescribing quality were examined using Poisson regression, where a two-sided *p*-value < 0.05 was considered significant. Number of prescriptions by category of prescribing quality was utilized as the dependent variable, with annual cohort population included as the offset. Post-hoc analyses included the frequency of duration (days) prescribed for first-line agents, and the relative proportions of “not recommended” and “clinically appropriate” by major anatomical therapeutic chemical (ATC) class.

## Results

**Fig 1:**
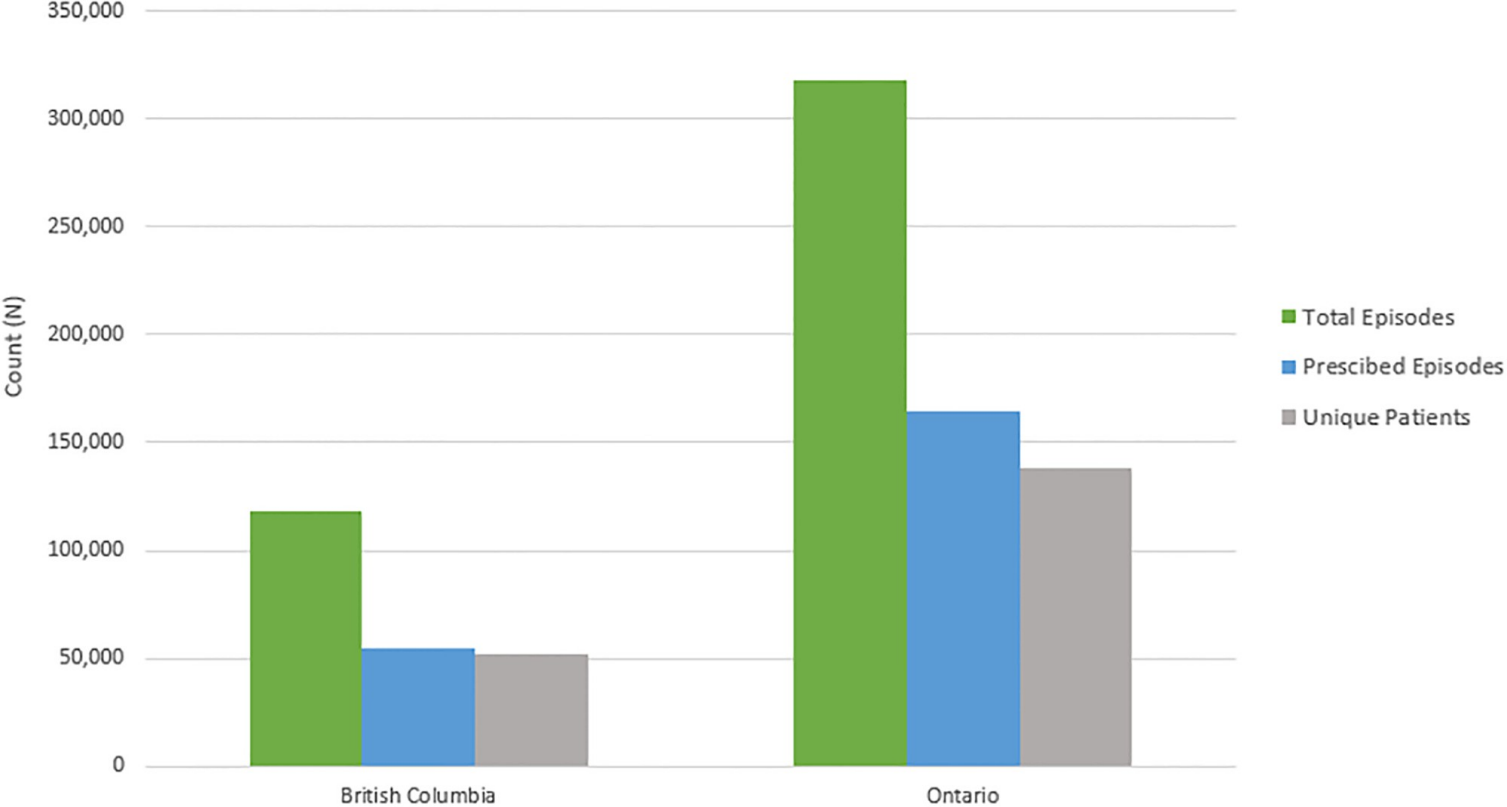
Counts of CAP episodes, prescribed episodes and unique patients.

Over the study period 118,606 and 317,823 total episodes of CAP were identified in BC and Ontario, respectively (Table 1). Of these episodes, 46% were prescribed an antibiotic in BC, and 52% in Ontario, resulting in 219,480 total episodes of CAP issued antibiotics (Fig 1). The average age for BC patients was 78 years and 77 years in Ontario, with slightly more females for both provinces (BC: 53.3%; ON: 55.1%). In both provinces, the overall rate of CAP episodes was 28 cases per 1000 population. The average number of physician visits per episode was 1.75 and 1.35, per province respectively. These episodes can be attributed to 51,981 unique patients in BC, and 138,157 in Ontario.

**Table 1:** Cohort characteristics.

	British Columbia	Ontario
**Total Episodes of CAP**^1^ **(N)**	118,606	317,823
**Prescribed Episodes of CAP (N)**	54,782	164,698
**Age**		
Mean ± SD	77.98 ± 8.37	76.97 ± 7.85
Median (IQR)	77 (71–84)	76 (70–83)
65–79 years	32,533 (59.4%)	105,429 (64.0%)
≥80 years	22,249 (40.6%)	59,269 (36.0%)
**Sex**		
Female	29,217 (53.3%)	90,830 (55.1%)
Male	25,565 (46.7%)	73,868 (44.9%)
**Income Quintile**		
Missing^2^	579 (1.1%)	437 (0.3%)
1	11,679 (21.3%)	34,536 (21.0%)
2	11,538 (21.1%)	34,509 (21.0%)
3	10,684 (19.5%)	33,022 (20.1%)
4	9,993 (18.2%)	30,710 (18.6%)
5	10,243 (18.7%)	31,484 (19.1%)
**Rurality**		
Missing	1,651 (3.0%)	371 (0.2%)
No	43,572 (79.5%)	141,655 (86.0%)
Yes	9,559 (17.4%)	22,672 (13.8%)
**Patient Comorbidities N (%) yes**		
Interacting Drugs^3^	333 (0.6%)	634 (0.4%)
Immunosuppressive Medications^4^	13,654 (24.9%)	30,250 (18.4%)
Diabetes^5^	21,203 (38.7%)	54,765 (33.3%)
Congestive Heart Failure^5^	8,548 (15.6%)	29,133 (17.7%)
Myocardial Infarction	4,981 (9.1%)	4,036 (2.5%)
COPD^5^	18,292 (33.4%)	65,048 (39.5%)
Asthma^5^	5,851 (10.7%)	42,433 (25.8%)
Coronary Artery Disease	8,788 (16.0%)	25,834 (15.7%)
Hypertension^5^	38,907 (71.0%)	135,751 (82.4%)
Cancer^4^	20,576 (37.6%)	71,755 (43.6%)
Chronic Kidney Disease	8,902 (16.3%)	18,215 (11.1%)
**Charlson Comorbidity Index**		
0	29,737 (54.3%)	132,431 (80.4%)
1–2	21,027 (38.4%)	20,503 (12.4%)
3–4	2,815 (5.1%)	8,390 (5.1%)
5+	1,161 (2.1%)	3,374 (2.0%)
**Visits in Episode (N)**		
Mean ± SD	1.75 ± 1.76	1.35 ± 1.02
Median (IQR)	1 (1–2)	1 (1–1)

^1^Acute episodes defined as all recurrent physician visits for CAP within 14 days (< 30 days)

^2^Missing represents absent or not applicable patient demographic information;

^3^Moderate/severe drug interactions for first-line CAP agents: amoxicillin, amoxicillin clavulanate, doxycycline;

^4^Immunosuppressive medications include: anti-rheumatic drugs, oral glucocorticoids, anti-rejection medication, and chemotherapeutic agents;

^5^Patient comorbidities present incident cases for each province with the following Ontario exceptions: diabetes, congestive heart failure, COPD, asthma, hypertension—which present prevalent cases due to data limitations (i.e., Ontario used any look back period whereas BC used a 1-year look back period due to data structure);

Abbreviations: BC—British Columbia, ON—Ontario, COPD—Chronic obstructive pulmonary disorder; SD—Standard deviation, IQR—Interquartile range, CAP—community-acquired pneumonia

### Category of prescribing quality

**Fig 2:**
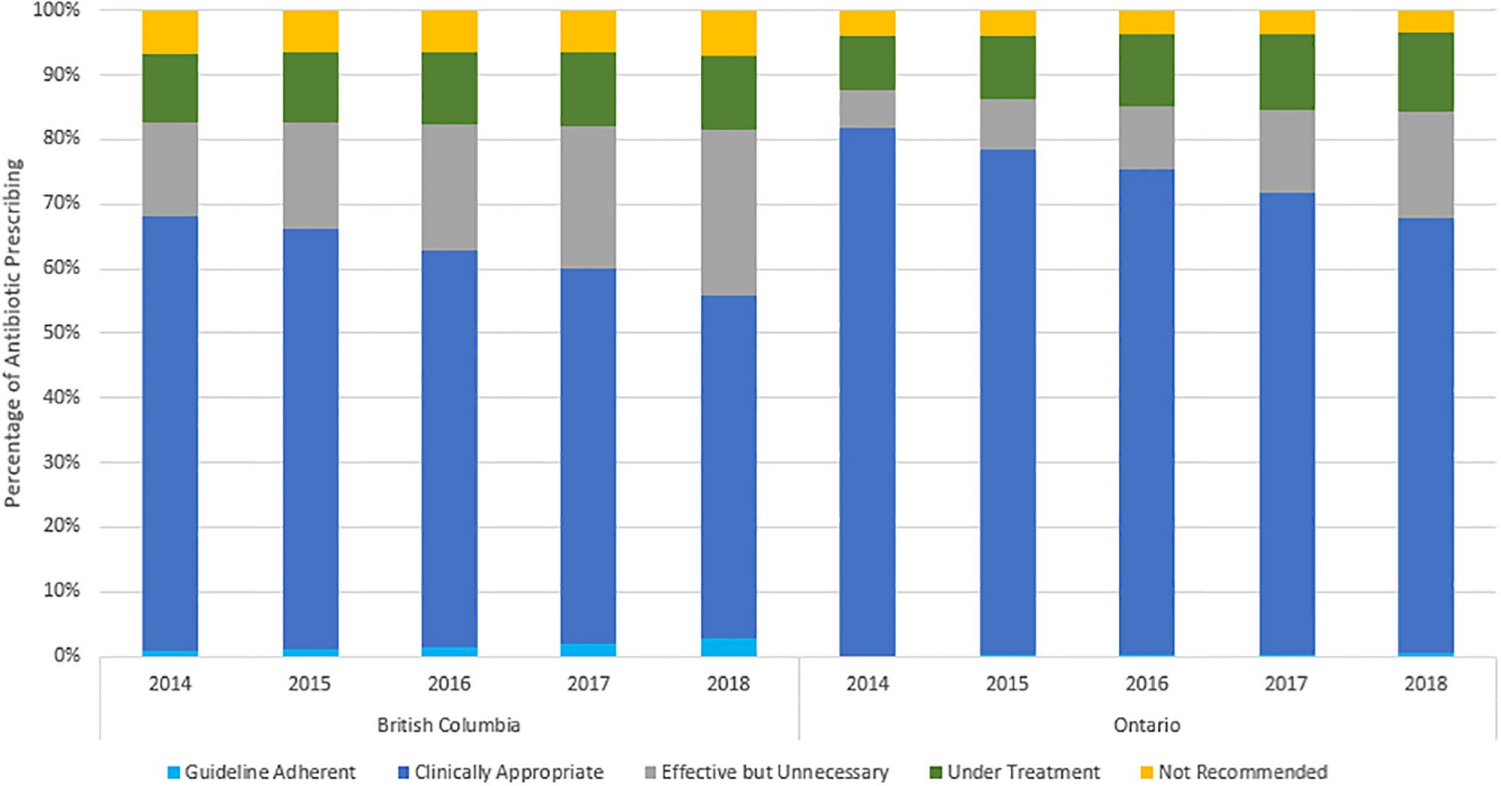
Percentage of outpatient antibiotic prescribing for CAP, by category of prescription quality and year.

**Fig 3:**
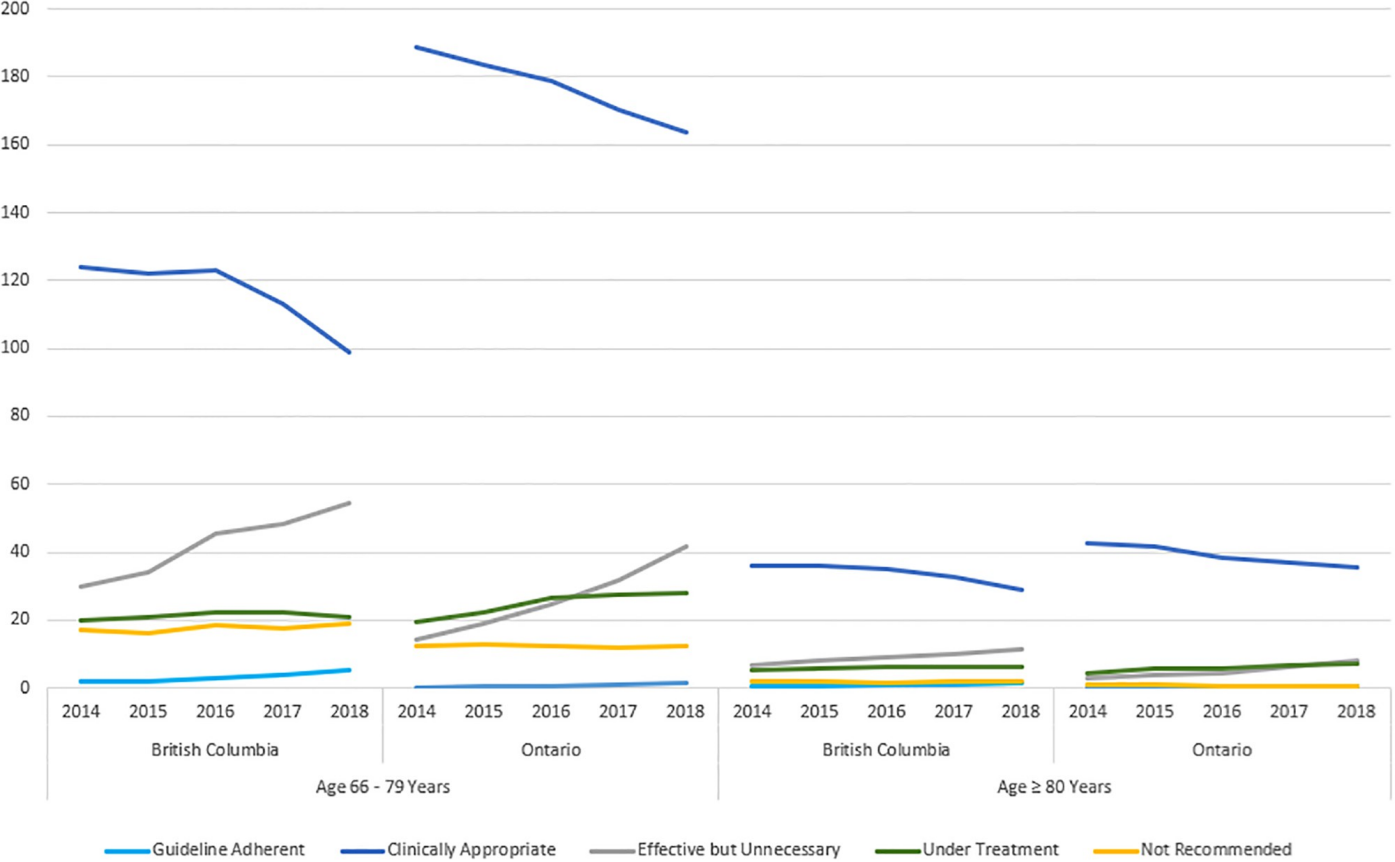
Rate of overall antibiotic prescribing for outpatient CAP in Canadian seniors, by age category and province.

Clinically appropriate prescribing accounted for the most antibiotics issued to older adults, for CAP, across all study years (BC: 60.7%; ON: 74.2%) (Fig 2). These prescriptions were mainly composed of non-first line agents dispensed in the presence of a clinical factor that could preclude the use of a guideline recommended agent (BC: 96.4%; ON: 97.9%). A complete list of these factors and their relevant cohort counts are available in Table 1. The remaining clinically appropriate prescriptions (BC: 3.6%; ON: 2.1%) can be attributed to the use of first-line agents, in the presence of chronic kidney disease, re-classified from undertreatment. In both provinces, rates of clinically appropriate prescribing significantly decreased over the study period (Table 2; Fig 3). By 2018, a 20% (RR: 0.80; 95% CI: 0.77–0.83) reduction in BC, and 15% (Rate Ratio [RR]: 0.85; 95% Confidence Interval [CI]: 0.84–0.87) reduction in Ontario, was observed in clinically appropriate prescribing, when compared to the first study year (Table 2).

**Table 2:** Patterns of antibiotic prescribing by category of quality for outpatient CAP in Canadian seniors.

Prescribing Quality	Province	2014		2015		2016		2017		2018		Δ	Rate Ratio^3^ (95% CI)
		Rate^1^	N^2^	Rate	N	Rate	N	Rate	N	Rate	N		
Guideline Adherent^4^	BC	2.16	85	2.89	129	3.54	155	4.74	233	6.93	329	2.21	3.37 (2.67–4.30)
	ON	0.37	42	0.56	68	0.74	82	1.26	155	1.86	242	4.05	4.62 (3.37–6.49)
Clinically Appropriate^5^	BC	159.92	6561	157.89	6947	158.12	6859	145.55	7109	127.80	6010	-0.20	0.80 (0.77–0.83)
	ON	231.47	23671	224.87	24784	217.43	23210	206.99	25367	199.10	25213	-0.14	0.85 (0.84–0.87)
Effective but Unnecessary^6^	BC	36.50	1414	42.34	1774	54.26	2185	58.71	2695	65.82	2913	0.80	1.79 (1.68–1.91)
	ON	16.87	1680	22.78	2478	29.04	3017	37.72	4524	49.66	6159	1.94	2.94 (2.78–3.10)
Under Treatment^7^	BC	25.10	1012	26.66	1164	28.59	1233	28.51	1375	26.88	1275	0.07	1.10 (1.01–1.19)
	ON	24.19	2479	28.00	3138	32.21	3436	34.11	4247	34.94	4541	0.44	1.47 (1.40–1.54)
Not Recommended^8^	BC	19.20	670	18.27	693	20.26	731	19.56	807	20.81	812	0.08	1.05 (0.95–1.17)
	ON	13.27	1143	13.60	1259	12.97	1147	12.48	1286	12.72	1330	-0.04	0.93 (0.86–1.01)

^1^age-standardized rate of prescribing per 1000 population;

^2^number of antibiotic prescriptions;

^3^rate ratios presented compare prescribing in 2018 against 2014;

^4^guideline adherent: concordant to first-line agent, dose and duration;

^5^clinically appropriate: discordant to first-line agent in presence of patient comorbidity, immunosuppressant medication and/or interacting medication;

^6^effective but unnecessary: concordant to first-line agent but dose and/or duration discordant;

^7^under treatment: concordant to first-line agent but dose and/or duration discordant;

^8^not recommended: discordant to first-line agent in absence of patient comorbidity, immunosuppressant medication and interacting medication;

Abbreviations: Δ—percentage difference between 2014 and 2018; BC—British Columbia; ON—Ontario

Guideline adherent reported the smallest proportions of antibiotic prescription in each study year, for both provinces (BC: 1.69%; ON: 0.36%) (Fig 2). These prescriptions were fully concordant by agent, dose *and* duration. Despite a two-fold and four-fold increase by 2018, in BC and ON respectively, guideline adherent prescribing remained the least prescribed category of antibiotic use (BC: 6.93 prescriptions per 1000 population; ON: 1.86 prescriptions per 1000 population) (Table 2; Fig 3).

Effective but unnecessary prescriptions, which were adherent by agent but excessive by dose and/or duration were the second-most identified category; accounting for 19.9% of all prescribing in BC, and 10.8% in Ontario (Fig 2). Although both provinces demonstrated increasing rates over time, “effective but unnecessary” prescribing was roughly double in BC, across all study years (Table 2; Fig 3). By 2018, when compared to the first study year, Ontario (RR: 2.94; 95% CI: 2.78–3.10) experienced a greater increase than BC (RR: 1.79; 95% CI: 1.68–1.91) in this category. Excess duration of therapy was the hallmark characteristic for effective but unnecessary prescribing categorization (BC: 92%; ON: 99%). The remaining effective but unnecessary prescriptions (BC: 8%; ON: 1%) were also excessive in treatment length, in addition to a dose beyond the average daily recommendation.

Undertreatment prescriptions were also adherent in agent, but subtherapeutic by dose and/or duration. This category followed as third most identified in both provinces, and as most comparable across provinces for magnitude of overall antibiotic utilization (BC: 10.98%; ON: 10.83%) (Fig 2). In Ontario, “under treatment” prescribing increased year-on-year with 35 prescriptions issued per 1000 population, by 2018. Contrastingly, BC “under treatment” antibiotic use peaked in 2016, before trending down in later study years with 35 prescriptions issued per 1000 population by 2018 (Table 2). Across both provinces, the majority of “under treatment” prescriptions (ON: 94%; BC: 79%) can be attributed to subtherapeutic dosing in the presence of excessive length of therapy. In BC, much of the remaining prescriptions are tied to either short duration of therapy (9%), subtherapeutic dosing (5%), or both (6%); while the residuals in Ontario are split evenly (2%) across the same categories.

Not recommended prescribing was the only category of prescribing to remain stable over time, in both provinces (BC—RR: 1.05; 95% CI: 0.95–1.17; ON—RR: 0.93; 95% CI: 0.86–1.01). These prescriptions were composed of non-guideline adherent antibiotic agents, in the absence of *all* clinical factors that would have precluded their use. Overall, “not recommended” antibiotic use was higher in BC (7%) than Ontario (4%), and this was true across each study year as well.

### Antibiotic classes and duration of therapy

In both provinces, clinically appropriate prescribing was comprised of mainly quinolones, then macrolides, and other beta-lactams, when examined by major ATC class. Macrolides accounted for the majority (BC: 42%; ON: 62%) of not recommended prescribing, followed by quinolones (BC: 35%; ON: 31%), and then other beta-lactams (BC: 11%; ON: 8%). S1 Fig contains the full summary for relative proportions prescribed for both categories across provinces.

With respect to the frequency of duration prescribed for first-line agents, (i.e. amoxicillin, amoxicillin clavulanate, doxycycline) 7 days was the most commonly dispensed across all agents, and ages (BC: 54%; ON: 49%). The second most common length of therapy was 10 days, followed by 5. S2 Fig shows the relative frequencies by province, patient age, and first-line agent.

## Discussion

This is the first study to evaluate appropriate prescribing for outpatient CAP, by agent, dose and duration. Our study found that “guideline adherent” prescribing, which are concordant with first line agents and recommended dose and duration, accounted for only 2% of prescriptions in BC, and less than 1% in Ontario. However, the majority of prescribing in both provinces was deemed “clinically appropriate”, wherein comorbidities justified the use of non-first line agents (BC: 61%; ON: 74%). The breakdown of appropriate prescribing between guideline adherent and clinically appropriate may be attributable to the study population—as older adults may present more routinely with comorbidities, requiring deviations from guideline adherent therapies. Not recommended prescribing was minimal in both provinces, with 4% of all prescriptions in Ontario, and 7% in BC, discordant with guidelines in the absence of any patient clinical justifications. Clinical practice guidelines, and literature preceding our study period provide evidence that shorter treatments are non-inferior for CAP [8–10, 23–25]. Regardless, duration was the leading determinant for effective but unnecessary prescription, with excessive lengths of therapy (≥7 days).

Given the highest incidence rates for CAP are amongst older adults (≥65 years), and the positive association between increasing age and risk, ensuring judicious use of antibiotics for this population is paramount. Multiple studies have also identified associations between increased in-hospital mortality and therapies used for a preceding episode of community acquired pneumonia [33, 34]. Chakrabarti et al. report that pre-treatment with antibiotics for CAP remain an independent risk factor for increased in-hospital mortality (Odds Ratio [OR]: 1.43, 95% CI: 1.19–1.71) after adjusting for age, and various comorbidities. This finding however may simply reflect a lack of response to previous empiric oral therapy being a poor prognostic factor. Johnson et al. found that 75% of antibiotics issued in the community, prior to a subsequent hospitalization for pneumonia, were appropriate, with 98% of these prescriptions reporting adequate dose, but with no additional analysis of duration of therapy. Appropriate prescribing prior to hospitalization conferred a 38% relative mortality reduction for patients. With respect to these data, our study found high rates of appropriate prescribing for CAP, which is favorable for Canadian older adults, in the event of subsequent hospitalization.

First line agents for this study included: amoxicillin, amoxicillin clavulanate and doxycycline. In BC, doxycycline accounted for a much larger proportion of prescriptions issued (43%), when compared to Ontario (4%). Previous work by this team had identified a provincial difference in tetracycline prescription, with BC using six times more than Ontario [35]. A potential explanation for this inter-provincial difference may be the shift to tetracyclines as a preferred agent in BC clinical resources, while beta-lactams remain primary agents in Ontario. With respect to non-first line agents, both the categories of clinically appropriate, and not recommended were examined by major ATC class. Macrolides and quinolones were the most selected classes of antibiotics for both categories. Macrolides were the most prescribed class of antibiotic in the *absence* of clinical justifications (i.e., not recommended antibiotic use). In BC, 48% of all not recommended prescribing were macrolides, with 62% in Ontario. Although macrolides are recommended alternatives in the presence of patient comorbidities—our study identified increased prescription in the absence of a host of patient factors, as listed in Table 1. Moreover, the use of beta-lactam monotherapy has been determined to be non-inferior to beta-lactam macrolide combination treatment [36]. Positively, the prescribing of quinolones and macrolides for outpatient treatment of community acquired pneumonia, in the absence of patient clinical justifications, accounts for a minority of overall prescribing in both provinces (BC: 6%; ON: 4%). It is possible that not recommended prescribing reflect antibiotic prescription in individuals with penicillin allergy. Although allergy data were unavailable for this study, reported population rates for beta-lactams are approximately 1 to 5% and may explain the use of non-first line agents in the absence of other examined comorbidities [37, 38].

This study has limitations inherent to all retrospective studies using administrative health data. Foremost, the absence of lab data to confirm the presence of infections, and case identification based on physician billing and ICD-9 codes may be subject to misclassification bias. However, Canadian physician claims data have reported high positive-predictive values for the diagnosis of common respiratory infections [39]. Moreover, codes utilized to identify CAP did not eliminate non-bacterial pneumonia. In BC, a unique diagnostic code is available for viral pneumonia (ICD-9: 480) however, available OHIP codes do not distinguish on etiology. As such, in order to ensure internal validity, all available pneumonia codes were included across both provinces and our reduced proportion of episodes prescribed (BC: 46%, ON: 52%) is likely attributable to the inclusion of cases that do not warrant antibiotic therapy. As only CAP-related codes were utilized in defining episodes of infection, and did not account for the potential of concurrent diagnoses, which may impact patient and prescribing misclassification. The absence of laboratory results led to a blanket assumption of non-atypical infection. The use of chronic kidney disease as a proxy for unknown creatinine clearance could also have underestimated our proportion of clinically appropriate prescribing, reclassified from under treatment. In a validation of Canadian outpatient data to determine chronic kidney disease, Fleet et al. identified only 33% sensitivity in older adults [40]. As such, it may be that the reported 4% (BC) and 2% (ON) attributed to the use of first-line agents, in the presence of chronic kidney disease re-classified from undertreatment, underestimates the true proportions. Allergy data was also unavailable and limited our characterization of non-first-line antibiotic prescribing. Current estimates report a range of 1–5% for beta-lactam allergies, and the absence of this data may have overestimated not recommended antibiotic prescriptions [37, 38]. With respect to antibiotic prescribing, unfilled prescriptions are not included within records, and our discussion of antibiotic use may be an underrepresentation of true provincial magnitude. Furthermore, prescription parameters of dose and duration were pulled from dispensation records, with no knowledge of patient adherence and/or presence of physician instructions (e.g. wait and watch stewardship efforts). Future research efforts should also engage with laboratory data to identify cases of *atypical* infection to elucidate elevated rates of macrolide and quinolone use.

## Conclusion

Community acquired pneumonia in older adults confers increasing risk for mortality, or functional status. Understanding the quality of antibiotic prescribing in this population is integral to maximize positive patient outcomes, while minimizing antibiotic-related adverse events, and the impact of prescribing on rates of bacterial resistance. This is the first study to review the quality of prescriptions issued for CAP, by agent, dose and duration, with the majority of antibiotic prescription clinically appropriate. However, the continued use of beta-lactam penicillins in place of macrolides or quinolones, and encouraging 3 to 5-day shorter course treatments present a clear direction for future stewardship efforts.

## Supporting information

S1 Table Category of prescription quality. (DOCX)

S2 Table Clinical factors underlying clinically appropriate antibiotic use. (DOCX)

S1 Fig Relative proportion of antibiotic use by ATC class for clinically appropriate and not recommended prescribing categories. (DOCX)

S2 Fig Frequency of duration prescribed by first-line agent, and patient age. (DOCX)

S1 Checklist STROBE statement—Checklist of items that should be included in reports of observational studies. (DOCX)
