# Supplementary material for: Retrospective cohort analysis of outpatient antibiotic prescribing for community-acquired pneumonia in Canadian older adults
Source: PLoS One. 2023 Oct 13;18(10):e0292899. doi: 10.1371/journal.pone.0292899 (PMC10575505; doi:10.1371/journal.pone.0292899)
Supplement: S1 Table — (DOCX) [file pone.0292899.s001.docx]

Supplement Table 1. Category of prescription quality

| **Prescription Quality** | **Definition** |
| --- | --- |
| **Guideline Adherent** | Correct drug *(i.e. first line agent)*, dose, duration according to prescribing guidelines for specific indication |
| **Under Treatment** | Use of antibiotic *(i.e. first line agents)* that can be improved in one of the following categories: (1) drug dose (*low* in comparison to guideline recommendation); (2) duration of therapy (*short* in comparison to guideline recommendation) in the absence of renal dysfunction   - Dose low; Duration guideline adherent - Dose guideline adherent; Duration short - Dose low; Duration short - Dose low; Duration long - Dose high; Duration short |
| **Effective but Unnecessary** | Use of antibiotic *(i.e. first line agents)* that can be improved in one of the following categories: (1) drug dose (*high* in comparison to guideline recommendation); (2) duration of therapy (*long* in comparison to guideline recommendation)   - Dose high; Duration guideline adherent - Dose guideline adherent; Duration long - Dose high; Duration long |
| **Clinically Appropriate** | Incorrect drug (*i.e. non-first line agent*) according to guidelines for specific indication but *at least one* of the following criteria met: (1) high-risk patient; (2) presence of comorbidity; (3) drug interactions preclude first line agent   - High risk patient status defined as presence of anti-rheumatic drugs, oral glucocorticoids, anti-rejection medication, and/or chemotherapeutic agents - Comorbidities included: diabetes, congestive heart failure, myocardial infarction, COPD, asthma, coronary artery disease, hypertension, cancer, chronic kidney disease, moderate/severe Charlson index score - Drug interactions included moderate/severe interactions only   Use of first line agent classified as undertreatment in the presence of renal dysfunction (i.e. chronic kidney disease) |
| **Not Recommended** | Incorrect drug (*i.e. non-first line agent* according to guidelines for specific indication in the *absence of all* the following criteria met: (1) high-risk patient; (2) presence of comorbidity; (3) drug interactions preclude first line agent   - High risk patient status defined as presence of anti-rheumatic drugs, oral glucocorticoids, anti-rejection medication, and/or chemotherapeutic agents - Comorbidities included: diabetes, congestive heart failure, myocardial infarction, COPD, asthma, coronary artery disease, hypertension, cancer, chronic kidney disease, moderate/severe Charlson index score - Drug interactions included moderate/severe interactions only |
