# Supplementary material for: Retrospective cohort analysis of outpatient antibiotic prescribing for community-acquired pneumonia in Canadian older adults
Source: PLoS One. 2023 Oct 13;18(10):e0292899. doi: 10.1371/journal.pone.0292899 (PMC10575505; doi:10.1371/journal.pone.0292899)
Supplement: S2 Table — (DOCX) [file pone.0292899.s002.docx]

Supplement Table 2. Clinical factors underlying clinically appropriate antibiotic use

| **Patient Status** | **Definition** | | |  |
| --- | --- | --- | --- | --- |
| Immunocompromised Status | Presence of any of the following medications within 6 months prior to index date^1^: | | |  |
|  | - Abacavir | - Erlotinib HCl | - Methylprednisolone | |
|  | - Abacavir sulfate–lamivudine–zidovudine | - Estramustine disodium phosphate | - Methylprednisolone acetate | |
|  | - Abacavir–lamivudine | - Etanercept recombinant | - Mycophenolate mofetil | |
|  | - Abatacept recombinant | - Etravirine | - Mycophenolic acid sodium | |
|  | - Abiraterone acetate | - Everolimus | - Natalizumab | |
|  | - Adalimumab | - Fludarabine phosphate | - Nelfinavir mesylate | |
|  | - Alemtuzumab | - Fosamprenavir calcium | - Nevirapine | |
|  | - Anakinra recombinant | - Gefitinib | - Nilotinib | |
|  | - Atazanavir | - Glatiramer | - Pazopanib HCl | |
|  | - Auranofin | - Gold sodium thiomalate | - Peginterferon alfa 2A recombinant | |
|  | - Azathioprine | - Gold sodium thiomalate disodium | - Prednisolone sodium phosphate | |
|  | - Betamethasone acetate–betamethasone sodium phosphate | - Golimumab recombinant | - Prednisone | |
|  | - Bevacizumab | - Hydrocortisone | - Rilpivirine | |
|  | - Budesonide | - Hydrocortisone sodium succinate | - Ritonavir | |
|  | - Capecitabine | - Hydroxyurea | - Rituximab | |
|  | - Certolizumab pegol | - Imatinib mesylate | - Saquinavir mesylate | |
|  | - Cladribine | - Indinavir sulfate | - Sirolimus | |
|  | - Cortisone acetate | - Infliximab | - Sorafenib tosylate | |
|  | - Cyclophosphamide | - Interferon alfa 2B | - Stavudine | |
|  | - Cyclosporine | - Interferon beta | - Sunitinib malate | |
|  | - Cytarabine | - Interferon beta 1A | - Tacrolimus | |
|  | - Darunavir | - Interferon beta 1B recombinant | - Temozolomide | |
|  | - Dasatinib | - Lamivudine | - Tenofovir disoproxil Fumarate | |
|  | - Delavirdine mesylate | - Lamivudine–zidovudine | - Teriflunomide | |
|  | - Dexamethasone | - Lapatinib ditosylate | - Thioguanine | |
|  | - Dexamethasone sodium phosphate | - Leflunomide | - Tocilizumab recombinant | |
|  | - Didanosine | - Lenalidomide | - Tretinoin | |
|  | - Efavirenz | - Lomustine | - Triamcinolone acetonide | |
|  | - Emtricitabine–tenofovir disoproxil fumarate | - Lopinavir–ritonavir | - Ustekinumab | |
|  | - Enfuvirtide | - Methotrexate | - Vemurafenib | |
|  |  | - Methotrexate sodium | - Zidovudine | |
| Charlson Comorbidity Index | Presence of moderate (3-4) or severe (5+) Charlson index score, as calculated from all hospital records (ICD-10) up to 2 years prior to index date | | | |
| Diabetes Mellitus^2^ | Presence of applicable medication dispensation(s) *OR* at least 2 outpatient physician visits *OR* at least 1 hospital admission within 1 year of index date  ICD-9/OHIP: 250  ICD-10: E10; E11; E12; E13; E14 | | | |
| COPD | At least 1 hospital admission record *OR* 1 outpatient physician visit within 2 years of index date  ICD-9/OHIP: 491;492;496  ICD-10: J41; J42; J43; J44 | | | |
| Asthma | At least 1 hospital admission record *OR* 2 outpatient physician visits within 2 years of index date  ICD-9/OHIP: 493  ICD-10: J45 | | | |
| Congestive Heart Failure | At least 1 hospital admission record *OR* 1 outpatient physician visit within 1 year of index date  ICD-9/OHIP: 428  ICD-10: I50 | | | |
| Myocardial Infarction | At least 1 hospital admission record *OR* 1 outpatient physician visit within 1 year of index date  ICD-9/OHIP: 410  ICD-10: I21; 122 | | | |
| Coronary Artery Disease | At least 1 hospital admission record *OR* 1 outpatient physician visit within 1 year of index date  ICD-9/OHIP: 410; 411; 412; 413; 414; 429  ICD-10: I20; I23; I24; I25; Z955; Z958; Z959; R931; T822 | | | |
| Hypertension | Presence of applicable medication dispensation(s) within 6 months of index date;  At least 1 hospital admission record *OR* 1 outpatient physician visit within 2 years of index date  ICD-9/OHIP: 401; 402; 403; 404; 405  ICD-10: I10; I11; I12; I13; I15 | | | |
| Cancer | Presence of at least 1 hospital admission record *OR* 1 outpatient physician visit within 5 years of index date  ICD-9/OHIP: V10; 140; 141; 142; 143; 144; 145; 146; 147; 148; 149; 150; 151; 152; 153; 154; 155; 156; 157; 158; 159; 160; 161; 162; 163; 164; 165; 170; 171; 172; 173; 174; 175; 176;179; 180; 181; 182; 183; 184; 185; 186; 187; 188; 189; 190; 191; 192; 193; 194; 195; 196; 197; 198; 199; 200; 201; 202; 203; 204; 205; 206; 207; 208; 230; 231; 232; 233; 234  ICD-10: 800; 801; 802; C00; C01; C02; C03; C04; C05; C06; C07; C08; C09; C10; C11; C12; C13; C14; C15; C16; C17; C18; C19; C20; C21; C22; C23; C24; C25; C26; C30; C31; C32; C33; C34; C37; C38; C39; C40; C41; C43; C44; C45; C46; C47; C48; C49; C50; C51; C52; C53; C54; C55; C56; C57; C58; C60; C61; C62; C63; C64; C65; C66; C67; C68; C69; C70; C71; C72; C73; C74; C75; C76; C77; C78; C79; C80; C81; C82; C83; C84; C85; C86; C88; C90; C91; C92; C93; C94; C95; C96; C97; D00; D01; D02; D03; D04; D05; D06; D07; D09; Z850; Z851; Z852; Z853; Z854; Z855; Z856; Z857; Z858; Z859; 803; 804; 805; 807; 808; 809; 811; 812; 813; 814; 815; 816; 817; 818; 819; 820; 821; 822; 823; 824; 825; 826; 827; 828; 829; 830; 831; 832; 833; 834; 835; 837; 838; 839; 840; 841; 842; 843; 844; 845; 846; 847; 848; 849; 850; 851; 852; 853; 854; 855; 856; 857; 858; 860; 862; 863; 864; 865; 868; 869; 870; 871; 872; 873; 874; 876; 877; 878; 880; 881; 883; 884; 885; 889; 890; 891; 892; 893; 893; 894; 895; 896; 897; 898; 899; 900; 902; 904; 905; 906; 907; 908; 909; 910; 911; 912; 913; 914; 915; 917; 918; 919; 922; 923; 924; 925; 926; 927; 929; 931; 933; 936; 937; 938; 939; 940; 941; 942; 943; 944; 945; 946; 947; 948; 949; 950; 951; 952; 953; 954; 956; 958; 959; 959; 965; 966; 967; 968; 969; 970; 971; 972; 973; 974; 976; 980; 982; 983; 984; 985; 986; 987; 988; 989; 990; 991; 993; 994 | | | |
| Chronic Kidney Disease | Presence of at least 1 hospital admission record *OR* 1 outpatient physician visit within 5 years of index date  ICD-9: 403; 858  ICD-10: E102; E112; E132; E142; I12; I13; N08; N18; N19 | | | |
| Drug x Drug Interaction | Presence of a moderate/severe drug interaction with first-line agent within 3 months prior to index date | | | |
| *1 “index date” defined as the first physician visit within an episode of community-acquired pneumonia;* 2 diabetes was not further characterized between type 1 and type 2; *Abbreviations: COPD – chronic obstructive pulmonary disorder* | | | | |
