# Supplementary material for: Retrospective cohort analysis of outpatient antibiotic prescribing for community-acquired pneumonia in Canadian older adults
Source: PLoS One. 2023 Oct 13;18(10):e0292899. doi: 10.1371/journal.pone.0292899 (PMC10575505; doi:10.1371/journal.pone.0292899)
Supplement: S1 Fig — (DOCX) [file pone.0292899.s003.docx]

Supplemental Figure 1. Relative proportion of antibiotic use by ATC class for *clinically appropriate* and *not recommended* prescribing categories
