# Supplementary figures and images for: Retrospective cohort analysis of outpatient antibiotic prescribing for community-acquired pneumonia in Canadian older adults
Source: PLoS One. 2023 Oct 13;18(10):e0292899. doi: 10.1371/journal.pone.0292899 (PMC10575505; doi:10.1371/journal.pone.0292899)

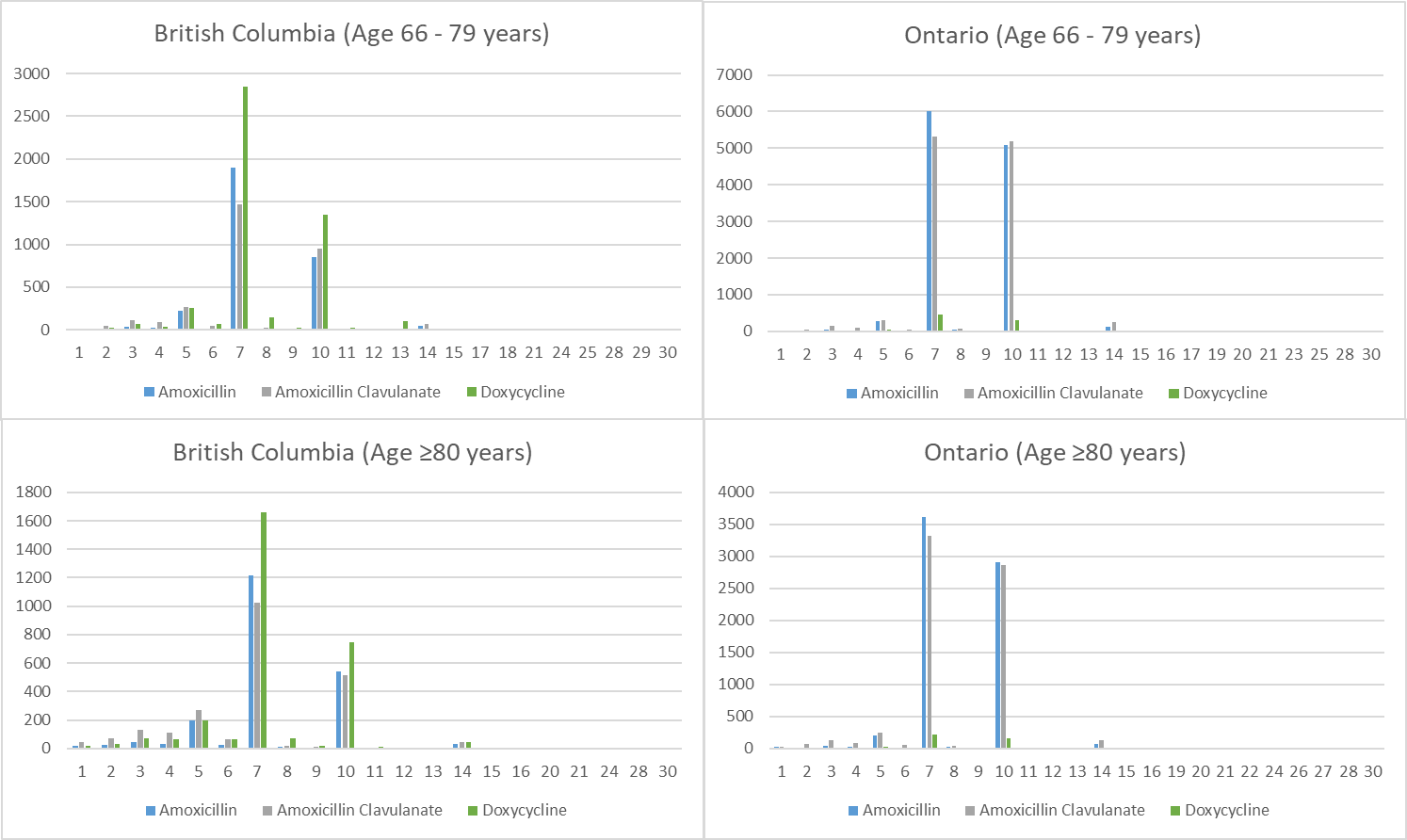
Supplemental Figure 2. Frequency of duration prescribed by first-line agent, and patient age

Supplement: S2 Fig — (DOCX) [file pone.0292899.s004.docx]
